# A systematic review defining non-beneficial and inappropriate end-of-life treatment in patients with non-cancer diagnoses: theoretical development for multi-stakeholder intervention design in acute care settings

**DOI:** 10.1186/s12904-022-01071-7

**Published:** 2022-11-09

**Authors:** Jamie Jay-May Lo, Nicholas Graves, Joyce Huimin Chee, Zoe Jane-Lara Hildon

**Affiliations:** 1 Saw Swee Hock School of Public Health and National University Health System, National University of Singapore, Tahir Foundation Building, 12 Science Drive 2, Level 09-03J, Singapore, S117549 Singapore; 2 Duke-NUS Medical School, 8 College Road, Singapore, S169857 Singapore

**Keywords:** Non-beneficial treatment, End-of-life care, Inappropriate treatment, Medical futility, Realist approach

## Abstract

**Background:**

Non-beneficial treatment is closely tied to inappropriate treatment at the end-of-life. Understanding the interplay between how and why these situations arise in acute care settings according to the various stakeholders is pivotal to informing decision-making and best practice at end-of-life.

**Aim:**

To define and understand determinants of  non-beneficial and inappropriate treatments for patients with a non-cancer diagnosis, in acute care settings at the end-of-life.

**Design:**

Systematic review of peer-reviewed studies focusing on the above and conducted in upper-middle- and high-income countries. A narrative synthesis was undertaken, guided by Realist principles.

**Data sources:**

Cochrane; PubMed; Scopus; Embase; CINAHL; and Web of Science.

**Results:**

Sixty-six studies (32 qualitative, 28 quantitative, and 6 mixed-methods) were included after screening 4,754 papers. Non-beneficial treatment was largely defined as when the burden of treatment outweighs any benefit to the patient. Inappropriate treatment at the end-of-life was similar to this, but additionally accounted for patient and family preferences.

Contexts in which outcomes related to non-beneficial treatment and/or inappropriate treatment occurred were described as veiled by uncertainty, driven by organizational culture, and limited by profiles and characteristics of involved stakeholders. Mechanisms relating to ‘Motivation to Address Conflict & Seek Agreement’ helped to lessen uncertainty around decision-making. Establishing agreement was reliant on ‘Valuing Clear Communication and Sharing of Information’. Reaching consensus was dependent on ‘Choices around Timing & Documenting of end-of-life Decisions’.

**Conclusion:**

A framework mapping determinants of non-beneficial and inappropriate end-of-life treatment is developed and proposed to be potentially transferable to diverse contexts. Future studies should test and update the framework as an implementation tool.

**Trial registration:**

PROSPERO Protocol CRD42021214137.

**Supplementary Information:**

The online version contains supplementary material available at 10.1186/s12904-022-01071-7.

## Background

There is evidence of the overuse of medical services globally, defined as the use of “medical services that are more likely to cause harm than good” [1]. Consequently, with the trends of ageing populations across middle- and high-income countries [2], concerns of “non-beneficial”, sometimes referred to as “medically futile”, treatment and determining the “inappropriateness” of such treatments at end-of-life have risen over the past decade [3, 4]. These terms are used in the literature to reference cases in which intensive curative treatment, usually delivered in acute care settings, may not be considered beneficial to a patient nearing end-of-life. However, what is meant by “beneficial” or “inappropriate” is fraught with ambiguity [4–6]. The perceptions across involved stakeholders of what makes a treatment “beneficial” or “inappropriate” require further defining [4, 7–11]. 

Although attempts have been made to capture prevalence of non-beneficial treatments, estimated in one review at 33–38% [9], much uncertainty surrounds its measurement. Clinical assessment tools have been argued to suffer unwarranted variation because they lack stakeholder (patients or caregivers, and clinicians) inputs [8]. Survey instruments are described as unstandardised, with wide variability in both content and administration [10]. Indeed, uncertainty relating to the definitions of non-beneficial treatment and affiliated constructs as well as the variability in guiding principles – both clinical and ethical – have been concluded in multiple reviews [4, 7, 11].

Nevertheless, the centrality of understanding the drivers in decision-making [12–15] and related communication [16–20], the importance of accounting for patient preferences [21–27], as well as the role of family [28–31] in influencing non-beneficial outcomes or appropriateness of treatment has been identified. Alongside these findings, targeted interventions to improve these and related outcomes, for example, the use of technology, or models of non-hospice care and advanced care planning are being evaluated [32–39].

In contrast, the development and consideration of multi-stakeholder intervention, theory-driven [40] design has had little attention. Similarly, much of the above cited areas of study have included a strong focus on patients with a cancer diagnosis, and much existing related knowledge is indeed specific to these patients alone [41–46], despite disparities between patients with and without a cancer diagnosis at the end-of-life being well documented [47, 48]. Focusing on patients with a non-cancer diagnosis allows in-depth synthesis of determinants countering or contributing to non-beneficial or inappropriate outcomes which are specific to this population. 

Accordingly, the current review draws from elements of Realist Principles [49, 50], focusing on the main concept of “What works for whom in what circumstances”. It draws on the central Realists constructs, known as Contexts, Mechanisms, and Outcomes (C-M-O). Contexts refers to the broader conditions and circumstances be they interpersonal, institutional, infrastructural, or cultural in which outcomes occur [50]. Mechanisms are defined as “reasoning, beliefs, feelings, motivations, and choices of individuals and groups, which lead to patterns of behaviour that we recognize as outcome” [50–53]. The Outcomes would be result, intentional or unintentional, produced from the Mechanisms based on varying Contexts [50].

Therefore, the aim was to review primary descriptive or explanatory studies for patients with a non-cancer diagnosis, in acute care settings, at the end-of-life with the C-M-O informed objectives of:

- Cataloguing and interpreting the definition of non-beneficial treatment and inappropriate treatment Outcomes at end-of-life;
- Elaborating the Contexts in which such Outcomes occur;
- Outlining the Mechanisms that are likely to contribute to or counter these Outcomes;
- Synthesising these findings into an evidence-based framework to guide intervention design in this patient sub-group.

To date, no such review has been undertaken. Utilizing a theory-driven approach [54] will allow end-of-life researchers and practitioners to beginning mapping the complexities of provision of multi-stakeholder interventions in patients at the end-of-life with a non-cancer diagnosis in acute care settings. Findings may ultimately be extended and tested in other patient groups and contexts.

## Methods

A systematic review and narrative synthesis was conducted, see PRISMA checklist (Supplementary file 1). Realist C-M-O principles [50, 55] were applied to frame the data extraction, and ultimately guide the analysis.

### Search strategy

Studies were retrieved from the following databases: Cochrane; PubMed; Scopus; Embase; CINAHL; and Web of Science. Recommendations for literature fitting the study criteria were also elicited from team members and extended networks working in palliative care.

The search string was developed with a senior librarian and was composed of text and/or MESH and thesaurus terms, adapted to selected databases, as described in Table 1. Searches were ran separately across all databases, and duplicates removed using electronic facility in EndNote [56], after the search results were compiled. Duplicates were removed manually from searches that were ran outside of EndNote compatible interfaces.

**Table 1:** Search string composition

	Search strings
Population	“Physician” OR “health practitioner” OR “patient” OR “family” OR “caregiver” terms AND
Concept	“Non-beneficial treatment” OR “medical futility” OR “inappropriate treatment” terms OR “ineffective care” terms AND
Outcomes	“Attitude to death” OR “definition” OR “experience” OR “perception” OR “service delivery” OR “decision-making” / “communication” terms

### Selection criteria

Peer reviewed studies, either qualitative, quantitative or mixed-methods were included if they focused on documenting service delivery in acute care hospital settings and experiences of non-beneficial and/or inappropriate treatment at end-of-life in upper-middle- or higher income and human development settings based on World Bank Classification [57, 58]. Countries were checked against the World Bank and Human Development Index (HDI) rankings. Middle- and lower-income countries were excluded due to having different priorities for end-of-life resource allocation and service delivery [59]. Relevant reviews were set aside for screening, none with the same objective as the current review were found.

Primarily, studies that considered solely patients with a cancer diagnosis alongside those focusing on chronic care management, such as hospice on community care models, as well as paediatric services were excluded. Studies with explicitly mixed populations consisting of patients with or without cancer diagnoses were however included, though only data relating to non-cancer diagnosis extracted where possible. Where findings were collapsed, patient profiles where reported were extracted to gauge as much as was feasible the degree to which patients with non-cancer diagnoses were represented. The same was done with papers with mixed oncology and non-oncology practitioner sampling. 

Exclusions were otherwise applied to studies evaluating clinical intervention or interventions for specific medical devices or non-beneficial treatment protocols, legal aspects of related decision-making, or inappropriate care not at the end-of-life. Additionally, all studies were quality appraised using the Quality Appraisal across Study Methods (QASM) checklist [60] and weak studies were excluded. The checklist considers the following dimensions: clarity of reporting; appropriateness of method to answer research question; validity of method of analysis; reliability of findings; and method specific criteria (scores range from 1–10 and are rated 1–4 for weaker studies; 5–7 for moderate; and 8–10 for strong).

Weak studies (QASM scores of < 5) were excluded on the rationale that they can dilute credible findings and to streamline extraction, interpretation and narrative reporting of findings. A phased, data-driven approach was applied to ensure inclusions were sufficiently current. First, title selections were limited to studies conducted in 2000 and beyond. However, at full-text screening, notable lags between data collection and publication, coupled with fast moving systems changes became apparent [61]. Inclusion criteria was then amended to studies conducted in 2005 onwards.

Lastly, non-English studies, case reports, protocols, and grey literature such as commentaries or dissertations were also excluded.

### Screening procedure

At the title screen, 10% of the literature were independently screened by two reviewers (JJL and ZJH), with 89% agreement. All disagreements were resolved through discussion. The remainder of the titles were screened by one reviewer (JJL) and cross-checked by a second one (ZJH). The two reviewers then double screened 20% of the abstracts, with 87% agreement. Disagreements were resolved through discussion and further refining of the exclusion and inclusion criteria.

The remaining abstracts and full text articles were screened by one reviewer (JJL), and any uncertainties were discussed and resolved through discussion. Full text articles were independently quality checked by two reviewers (JJL and JHC).

### Data extraction according to C-M-O

Extractions were compiled in Microsoft Excel. First, explicitly given definitions and/or study measurement approaches that operationalized the terms non-beneficial or medically futile treatments (observed to be used interchangeably) as well as inappropriate treatment Outcomes were extracted. Next, Contexts were extracted in which the Outcomes were occurring, such as commonly described circumstances, settings, etc. Lastly, findings related to Mechanisms evidenced to either contribute or counter the Outcomes were extracted. All extractions were tagged according to stakeholder, i.e. patients or family/caregivers or healthcare practitioners, perspectives.

### Data analysis, synthesis, and reporting

Coding frames were generated iteratively, grouping extractions according to stakeholder. Groupings were discussed and agreed among two analysts (JJL and ZJH) and summarised using a matrix method of reporting (objectives a-c, Tables 2, 3 and 4). A narrative synthesis leading to the development of a Framework showing the C-M-O pathways (objective d, Fig. 2) was undertaken. Top level findings, corresponding to the respective matrices are presented accordingly with total number of studies which contributed to the finding in brackets and illustrated with examples from the included literature. See Supplementary File 2 for all citations linking to references listed in superscripts within Tables 2, 3 and 4.

## Results

**Fig. 1:**
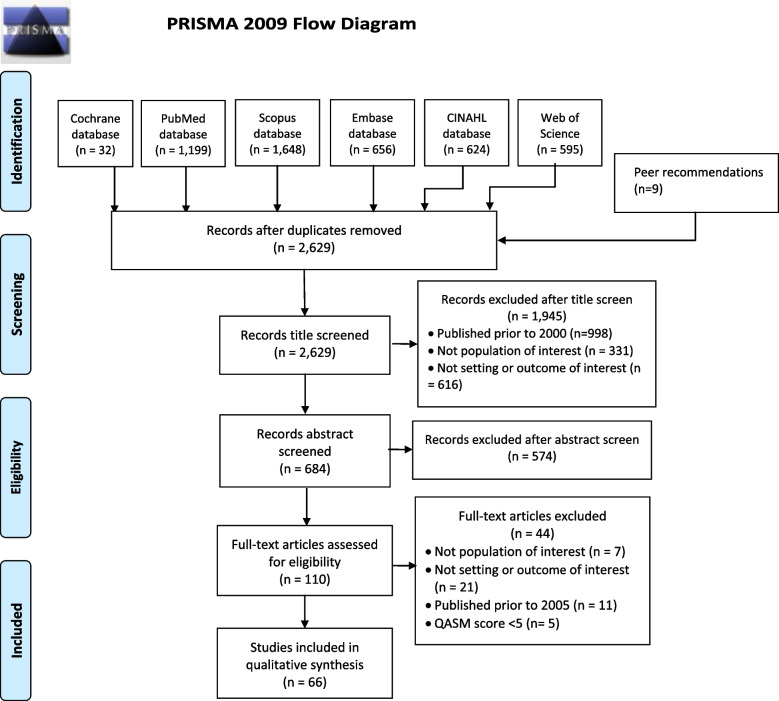
PRISMA 2009 flow diagram

The electronic database searches were run between March 15th to March 31st, 2020. These identified a total of 4,754 studies, and 2,629 studies after removal of duplicates and addition of articles from peer recommendations. After title, abstract, full-text, and quality check screening against inclusion criteria, 66 studies met the inclusion criteria (Fig. 1). Information of each included study is indicated in Supplementary File 2.

Forty-eight percent of included studies were published within the five years of the current searches (2015 to 2020). Included papers consisted of 32 qualitative studies, 28 quantitative and 6 mixed-methods studies, from 25 high- and middle-income countries. Most studies were from the United States of America (*n* = 21) and Australia (*n* = 10). Forty-one percent of studies had a QASM quality score rated “strong” (QASM scores between 8 to 10), and the remainder were rated “moderate” (QASM scores between 5 to 7). Five studies rated as “weak” (QASM scores less than 5) were excluded.

Mixed cancer / non-cancer patient population studies comprised 79% of the patient-related literature subset. In studies where findings were collapsed, reported sampling of cancer patients did not exceed 33%. Practitioner side analyses tended to sample from across hospital departments and thus 92% had a mix of specialist oncology/non-oncology and generalist populations. Oncology specialisations fell within less than a quarter of the reported samples.

- Cataloguing and interpreting the definition of non-beneficial and inappropriate treatment Outcomes at end-of-life (Table 2)

**Table 2:** Matrix of defining non-beneficial and related inappropriate treatment at end-of-life based on relevant review literature^a^

	Patients and family/guardians’ perspectives	Healthcare practitioners’ perspectives
**Alignment to Clinical Benefit or with Agreed Treatment Goals**		
**Non-beneficial treatment**	• ***Attempts at curative or life-prolonging treatments that are not consistent with the agreed goals of care***, i.e., especially when these are continued though unlikely to succeed, and/or considered by patient, family/guardians to result in unacceptable quality of life^1−19^ (*n* = 19)	• ***Burden likely to outweigh the clinical benefits of treatment***^2−4, 9−11, 14−15, 18−42^ (*n* = 32), i.e., would fail to achieve positive results such as recovery, symptom relief, or quality of life improvement • When curative treatment is no longer an option but ***continues or is escalated leading to excessive or aggressive care*** (*n* = 9) ^14, 17, 18, 22, 24, 25, 43−45^ ***inhibiting a peaceful death or prolonging the suffering of dying***^24, 44, 46, 47−49^ (*n* = 6)
**Inappropriate treatment at end-of-life**	• ***Treatment that are counter to the patient’s or family/guardians’ wishes can be deemed inappropriate***^5, 18, 19^ (*n* = 3)	• ***It can be deemed inappropriate to extend considerable resources when these are judged likely to exceed the clinical benefits or reasonable hop****e*^2, 31, 36, 40, 48^ (*n* = 5)

^a^Please see Supplementary File 2 for reference list as ordered in Table citations

### The need for alignment to clinical benefit or with agreed treatment goals

When there is a lack of alignment either to clinical benefit or with the agreed goals of care, treatments were generally defined as non-beneficial. “Inappropriate” end-of-life care was defined by accounting for patient preferences, protecting their wishes and even those of the family and patient’s inner circle, which speaks to the importance of the patient and family/guardians’ in agreeing goals of care.

### Non-beneficial treatment

The most repeated definition from the healthcare practitioners’ perspective related to when the burden of treatment outweighed the clinical benefits of treatment (cited in *n* = 32 studies, see Table 2 for list of citations). For example, a comprehensive survey of 688 health professionals in Canada found that 88% of respondents agreed with non-beneficial treatment being defined as “advanced curative/life-prolonging treatments that would most certainly result in a quality of life that the patient has previously stated that he/she would not want” [62]. Practitioners also extended this definition by referencing when a curative treatment was no longer an option but continues or is escalated leading to excessive or aggressive care (*n* = 9), inhibiting a peaceful death or prolonging the suffering of dying (*n* = 4).

From the patients and family/guardians’ perspectives curative or life-prolonging treatments that would not be consistent with the agreed goals of care (*n* = 19) were defined as non-beneficial. For instance, a qualitative study in Germany among 29 health professionals found that majority of participants associated non-beneficial treatment with “the lack of attainable goals of care” [63]. These were simply described as not improving prognosis or to result in unacceptable quality of life to the patient.

### Inappropriate end-of-life treatment

Inappropriate treatment were those that run counter to the patient’s or family/guardians’ wishes (*n* = 3). This is illustrated by an online survey with open questions among 592 patients and relatives in the Netherlands which found that following the patient’s, as well as relatives’ wish, was commonly mentioned to describe appropriate care [64]. It was also notable that it can be considered appropriate when patient or family/guardian understands that a treatment is unlikely to succeed but chooses to attempt a curative or life-prolonging treatment anyway [64–66] (*n* = 3). For instance, a survey in Japan among physicians and laypeople found that even if patients had sufficient information regarding a potential treatment, there was still a difference in judgements between patients and physicians due to different perspectives of the importance of medical information [65].

However, some clinicians may juggle or seek to find a balance between hope and lack of benefit, with a minority reporting that it may be judged appropriate to continue non-beneficial treatment to give hope [63, 64, 67] (*n* = 5). An example of this would be a qualitative study in Germany among clinicians, which found that a minority of the participants cited hope as a reason in providing futile treatment [63]. While others have argued that it is inappropriate to extend considerable resources when these are judged likely to exceed the clinical benefits or reasonable hope (*n* = 5). For instance, nurses working in an ICU from a qualitative study in Iran described futile treatment as “useless and ineffective care associated with waste of resources and torments of patients and nurses” [68].

b) Elaborating the Contexts in which non-beneficial and inappropriate treatment Outcomes at end-of-life occur (Table 3)

**Table 3:** Matrix based on relevant review literature^a ^ relating to contexts^b^ under which non-beneficial (NBT) and / or inappropriate end of life treatment can occur according to stakeholder perspectives^c^

**Uncertainty** Patients & family/ guardians	***Patient wishes unknown*** (*n* = 5) • Mismatch to patient goals/wishes, e.g. clinicians unsure what patient wants or patients not asked what their wishes are ^5, 19, 27, 37, 50^ (*n* = 5) ***Ambiguity in expectations*** (*n* = 9) • Difficulty with sharing prognosis or discordance in decision-making between relevant actors to support adhering to patient wishes ^22, 29, 32, 39, 42, 50−52^ (*n* = 8) • Family/guardians doubt physicians’ ability to predict NBT ^16^ (*n* = 1)
Healthcare practitioners	***Uncertainty of criteria for prognosis and related legal consideration*** (*n* = 37) • Lack of shared criteria for NBT guiding practitioners ^2−6, 8, 12, 13, 15−20, 22, 27, 28, 30−33, 35−37, 39, 44−46, 50, 52−59^ (*n* = 37); not being up to date on legal/policy for when to withdraw treatment ^3, 15, 28, 32, 46, 57^ (*n* = 6) ***Lack of shared understanding of duty of care*** (*n* = 12) • Duty of care means different things to different people e.g. “death as failure”, or adhering to moral duty to help provide a “good death” ^3, 18, 28, 30, 32, 35, 45, 48, 52, 55, 59, 65^ (*n* = 12)
**Organizational Culture & Practices** Patients & family/ guardians	***Emphasis on following directives*** (*n* = 4) • Removes emphasis from agreeing goals of care with the clinical team ^3, 23, 37, 49^ (*n* = 4)
Healthcare practitioners	***Practitioner inexperience and hospital level effects*** (*n* = 18) • Lack of emphasis on shared decision-making for NBT within clinical team across groups, leading to moral distress or burnout, especially among nurses ^12, 14, 33, 34, 41, 43, 44, 48, 53, 60^ (*n* = 10) • Practitioners are uncomfortable and unguided in how to deal with death and dying ^52^ (*n* = 1) • After adjusting for the role of patient and family role/directives it is shown that hospital and organizational cultural barriers are likely to contribute to NBT ^21^ (*n* = 1) • Organizational atmosphere and structure ^8, 45, 48^ (*n* = 3); lack of organizational support for dealing with NBT and assisting appropriate decision-making ^3, 26, 32, 36^ (*n* = 4); lack of promoting structure and function of hospital ethics committees^48^ (*n* = 1) ***Resource considerations*** (*n* = 10) • Resource implications of providing potentially NBT ^2, 29, 31, 36, 40, 48^ (*n* = 6) • Lacking access to and integration of palliative care, such as from palliative care centers or hospices ^10, 48, 50, 59, 62^ (*n* = 5)
**Profiles & Characteristics** Patients & family/ guardians	***Clinical presentation*** (*n* = 5) • Patient is more severely unwell ^19, 26^ (*n* = 2); emergency admissions to ICU, and a longer ICU or hospital stay ^19^ (*n* = 1); more elderly ^6, 21, 54^ (*n* = 3) ***Religious beliefs can underpin pushing for likely clinically NBT*** (*n* = 3) • Families and patients who are religious (Protestant, Catholic, Jewish) more frequently want more extensive treatment ^63^ (*n* = 1)
Healthcare practitioners	• Healthcare professionals with higher religiousness/spirituality more frequently tended to want to provide more extensive treatment ^47, 52, 63^ (*n* = 3) ***Differences were observed between cadres on views about withdrawing likely clinically NBT*** (*n* = 15) • Nurses differed in perception of NBT, especially on when it would be time to withdraw curative attempts, compared to doctors; often nurses or junior doctors placed higher importance in patient quality of life and functionality versus some doctors, especially senior doctors ^12, 14, 28, 32−34, 41, 43, 44, 47, 48, 53, 60, 61^ (*n* = 15)

^a^Please see Supplementary File 2 for reference list as ordered in Table citations

^b^The Contexts are defined as the broader conditions of the circumstances, including interpersonal, institutional, infrastructural, or cultural, etc. [50]

^c^See Table 2 for definitions of terms

### Uncertainty

Commonly non-beneficial and inappropriate treatment outcomes were shrouded in uncertainty. Indeed, over half of the included papers (56%) alluded to this construct. Largely, uncertainty was related to decision-making and perceptions of what constitutes a ‘benefit’ – affecting the different stakeholders involved in these processes in different ways. Uncertainty was typified when the patient wishes were unknown (cited in *n* = 5 studies, see Table 3 for list of citations), resulting in a potential mismatch of patient wishes to treatment goals. For instance, a qualitative study of nurses’ experiences, working over multiple ICUs over a mean of 7 years reported that the decision about whether to continue or halt treatment was unilaterally seen to be made by medical staff [69].

Ambiguity in expectations (*n* = 9) was caused by difficulty with sharing prognosis or discordance in decision-making between relevant actors to support adhering to patient wishes. As one study that surveyed ICU patients or their surrogates and practitioners showed, disagreement between patient side versus clinicians side regarding treating ‘too much’ occurred in 26% of cases, disagreement about treating ‘too little’ occurred in 10% of cases [70]. Correspondingly, disagreement about perceived inappropriate treatment was associated with prognostic discordance (*p* = 0.02) [70]. One study illustrated that ambiguity may emerge from family/ guardians doubting physicians’ ability to predict non-beneficial treatment [71].

However, by far, the biggest common finding across all included studies was reference to uncertainty of criteria for prognosis (*n* = 37), which extended to legal consideration (*n* = 6) such as not being up to date on legalities or policies for when to withdraw treatment. The former was documented to often result from a lack of shared criteria for non-beneficial treatment to guide practitioners. For example, a qualitative study in Australia found that there was uncertainty among medical teams as to the role of palliative care, and a discrepancy between medical and nursing views on how to administer palliative care [72]. 

A lack of shared understanding of duty of care (*n* = 12), or what the primary duty of a healthcare practitioner should be, also contributed to uncertainty. This was because the notion of duty meant different things to different people. For some, death was seen as “a failure” [73]. For others, they viewed the primary duty of a healthcare practitioner to be to provide a “good death” [73, 74]. 

### Organizational cultures and practices

The uncertainty of duty of care was further exacerbated by organizational cultures and practices that removes emphasis from agreeing goals of care from the patients and family/guardian with the clinical team (*n* = 4). For example, it was found that medical staff felt obligated to provide non-beneficial treatment at family requests despite knowing this may not be to the patients’ benefit. A survey among 333 clinicians across several hospitals in the USA found that 61% of participants attributed patient’s family as the main reason for providing non-beneficial treatment [75].

In addition, practitioner inexperience and hospital level effects (*n* = 18), which led to non-beneficial treatment, were defined by lack of emphasis on shared decision-making within clinical team across practitioner groups, leading to moral distress or burnout, especially among nurses. This moral distress and burnout was also influenced by the lack of leadership and practitioner support, which was described as entrenched by organizational atmosphere and structure. Resource considerations (*n* = 10), such as those implied in providing non-beneficial treatment as well as palliative care, were also found to be relevant contextual considerations for healthcare practitioners.

### Profiles and characteristics

Lastly, certain profiles and characteristics were found to be connected to contexts where non-beneficial treatment occurred. This manifested first and foremost in patients’ clinical presentation (*n* = 5). Those who were more severely unwell, who were emergency admissions and had longer ICU or hospital stays, and who were more elderly, were more likely to receive non-beneficial treatment. Such patients would also be more likely to have impaired ability for decision-making [64, 76]. In addition, religious beliefs can underpin pushing for likely clinically non-beneficial outcomes (*n* = 3). This was illustrated by a study examining the differences in end-of-life decisions from six European countries, which found that patients, families, as well as clinicians that were religious (Protestant, Catholic, Jewish) more frequently tended to want the more extensive treatments [77].

On the healthcare practitioner side, it was similarly shown that healthcare professionals with higher “religiousness/ spirituality” more frequently tended to want to provide the more extensive potentially non-beneficial treatments. For instance, an Australian qualitative study concluded that some clinicians had observed more religious doctors to be more likely to administer non-beneficial treatment [78]. More generally, differences were observed between practitioner groups (i.e. nurses, junior doctors, senior doctors) on views about withdrawing likely non-beneficial treatment (*n* = 15). Interestingly, nurses and junior doctors differed in perception of non-beneficial treatment compared to other doctors, especially senior doctors.

Nurses, for example, tended to place higher importance on patient quality of life and functionality versus doctors. This is discussed in a mixed-methods study carried out in Sweden, which found doctors were more likely to consider patient’s prognosis as a rationale for full life-sustaining treatment compared to nurses, who in turn prioritized the functional status and social circumstances of the patient (*p* < 0.01) [79]. Similarly, a survey-based study from Hungary demonstrated how subjective futility (i.e. whether a treatment was subjectively inappropriate vs. clinically non-beneficial) tended to be valued more among junior doctors compared to their seniors (*p* < 0.05); in addition, having more years of experience was associated with decreased perceived importance of the opinion of other medical staff [80].

iii) Outlining the Mechanisms that are likely to contribute to or counter non-beneficial and inappropriate treatment Outcomes at end-of-life (Table 4)

**Table 4:** Matrix based on relevant review literature^a^ relating to mechanisms^b^ that are likely to contribute to or counter decision-making for non-beneficial (NBT) and / or inappropriate end-of-life (EoL) treatment according to stakeholder perspectives^c^

	**Contributing mechanisms**	**Countering mechanisms**
**Motivation to Address Conflict & Seek Agreement** Patients & family/ guardians Healthcare practitioners	***Conflict and disputes*** (*n* = 14)	***Agreement seeking*** (*n* = 12)
	Conflict between practitioners and family on agreeing goals of care • Conflict between medical staff and family/guardians ^5, 19, 20, 21, 32^ (*n* = 5) • Conflict within the family ^21, 64^ (*n* = 2)	Getting patients and families to engage and seek consensus • Mandatory family meetings ^18^ (*n* = 1) • Patient’s wishes, explicitly sought ^13, 18, 19^ (*n* = 3)
	Conflict and lack of consensus seeking demonstrated across practitioner groups • Discord and hierarchical ‘pulling rank’, between doctors and nurses or between senior doctors and junior doctors on what constitutes NBT ^7, 18, 33, 43, 47, 53, 61, 65^ (*n* = 8)	Practitioners seeking consensus and collaboration • Environments or interactions promoting collaboration within medical team ^2, 12, 24, 40, 41, 43, 58, 61^ (*n* = 8) • Reaching consensus across practitioner groups with multi-disciplinary meetings ^36, 58^ (*n* = 2)
**Valuing Clear Communication and Sharing of Information** Patients & family/ guardians	***Misunderstandings and stonewalling*** (*n* = 13)	***Sharing information well*** (*n* = 8)
	Misunderstanding of treatment and prognosis • Problem of not clearly communicating treatment and prognosis to lay people/patients ^5, 10, 19, 22, 25, 29, 37, 42^ (*n* = 8) Presence of medical paternalism • Lack of sufficient communication between clinicians and patient or family/guardian, such that agreement on goals of care is curtailed ^5, 20, 27, 29, 43, 46, 50^ (*n* = 7)	Public awareness and education • Awareness and education of what constitutes NBT and appropriate decision-making at EoL ^5, 36, 37^ (*n* = 3)
Healthcare practitioners	Poor communication within medical team • Lack of collaborative decision-making within medical team across groups (i.e. nurses, junior doctors, senior doctors) ^12, 14, 33, 43, 48, 60^ (*n* = 6)	Promoting ongoing dialogues among practitioners • Engaging practitioners in formal and informal communication on NBT ^1, 3, 5, 18, 51, 52^ (*n* = 6)
**Choices around Timing & Documenting of EoL Decisions** Patients & family/ guardians Healthcare practitioners	***Suboptimal timings and communication*** (*n* = 8)	***Planning ahead well*** (*n* = 4)
	Engaging too late to agree goals of care, with inadequate communication tools • Discussions regarding EoL decision-making are delayed or stalled ^10, 13, 50, 66^ (*n* = 4)	Documenting patient decisions • Improved advanced care planning, do not resuscitate and goals of care documentation ^1, 13, 50, 64, 66^ (*n* = 5)
	Fixed directives that don’t account for changing circumstances • Can oblige the practitioner to carry on with NBT that may be causing suffering in incapacitated patients ^3, 23, 48, 64^ (*n* = 4)	Consolidation and streamlining of care • Having a team leader or primary clinical point-of-contact (i.e. specialist nurse) to oversee decision-making process relating to NBT across entire hospitalization ^10, 50^ (*n* = 2)

^a^Please see Supplementary File 2 for reference list as ordered in Table citations

^b^Mechanisms are defined as “reasoning, beliefs, feelings, motivations, and choices of individuals and groups, which lead to patterns of behavior that we recognize as outcome” [50–53]

^c^See Table 2 for definitions of terms

### Motivation to address conflict & seek agreement

Mechanisms relating to motivation to address conflict and seek agreement were important, since they helped to assuage uncertainty around decision-making; wanting to seek agreement was a starting point to addressing uncertainty and bridging differences of opinion.

Conflicts and disputes (cited in *n* = 14 studies, see Table 4 for list of citations) occurred between and across clinical and non-clinical parties. Conflicts between medical staff and family/guardians, or within the family on agreeing goals of care, were documented to affect treatment outcomes. Discord within the medical team was found to be coupled with hierarchical “pulling rank” between doctors and nurses, or between senior doctors and junior doctors. For example, a study in the United States explained how nurses were “walking a fine line” to avoid verbal reprimand by the physician. This manifested when nurses felt changing the patient’s code status to palliative care was warranted – ‘‘Sometimes they [physicians] just chop you at the legs if you mention something like that [change of code status]’’ [81].

Mechanisms that focused on agreement seeking (*n* = 12) were therefore pivotal to countering non-beneficial treatment or inappropriate outcomes. The value of getting families to engage and seek consensus, through mandatory family meetings, or through explicitly seeking patient’s wishes was likewise notable. Similarly, environments or interactions promoting collaboration among the medical teams, such as being open to nurses’ feedback, and those that explicitly aimed at reaching consensus across practitioner groups with multi-disciplinary meetings also countered poor treatment outcomes. This mechanism is illustrated by confirmed poorer outcomes (higher hospital mortality) when assessments of non-beneficial treatment were shared between doctors and nurses and consensus on this was reached, rather than when non-beneficial treatment was assessed solely by the nurse or the doctor [82].

### Valuing clear communication and sharing of information

In terms of valuing clear communication and sharing of information, misunderstandings and stonewalling (*n* = 13) contributed to inhibiting stakeholders from reaching agreement and consensus. The problem of not clearly communicating treatment and prognosis to lay people/patients was notable. For instance, a qualitative study among family members of terminal ICU patients in Brazil, highlighted that unsatisfactory communication stemmed from the lack of clarity, objectivity, and emotional preparedness of clinicians when communicating with family members [83]. Ineffective communication can lead to medical paternalism; meaning there is a lack of sufficient communication from clinicians such that agreement in goals of care and appropriate treatment, from the patient and family/guardian perspectives, can be seen to be impeded.

In echo, a survey among laymen in Japan found that 75% of participants believed that clinicians should inform the patient/family if non-beneficial treatment was likely to occur, but the final decisions should be from the patient [84]. From the healthcare practitioner perspective, poor communication within the medical team, leading to a lack of collaborative decision-making, was noted to contribute to the provision of non-beneficial treatment. In contrast, mechanisms that promoted sharing information well (*n* = 8) across stakeholders helped counter medical paternalism and its effects. This worked by flattening hierarchies through deliberate strategies to promote ongoing dialogues among practitioners, for instance engaging in formal and informal communication through mentorship and discussion forums for physicians [78].

It was also demonstrated by Anstey et al. [73] whose study of ICU practices in California found that 90% of clinician participants (95% CI: 88% to 92%) supported formal communication training to prevent non-beneficial treatment or inappropriate outcomes. Similarly, increasing public awareness and education of what constitutes non-beneficial treatment and appropriate decision-making at end-of-life will render these topics more transparent and less taboo. To illustrate this gap, a study in Canada surveying healthcare professionals connected the lack of public awareness and preparedness for end-of-life decision-making to one fifth of caregivers not knowing – and thus not being able to help address – the patient’s wishes [85].

### Choices around timing and documenting of end-of-life decisions

Building on these findings, choices around timing & documenting of End-of-Life Decisions were also found to be important to countering non-beneficial treatment or inappropriate outcomes. Suboptimal timings and communication (*n* = 8) were often times due to engaging patients and their families too late to agree on goals of care with inadequate communication tools, therefore delaying or stalling end-of-life decision-making discussions. For instance, as found in a study from the United States, delays and stalling in decision-making greatly increased the association with a patient receiving inappropriate treatment (OR 4.52, 95% CI: 1.69–12.04, *p* = 0.003) [86]. Another facet of suboptimal timings for discussions and documentations would be fixed directives that do not account for changing circumstances. For example, it was found that almost two thirds (64%) of patients had changed their end-of-life care preferences at least once over two years [87].

**Fig. 2:**
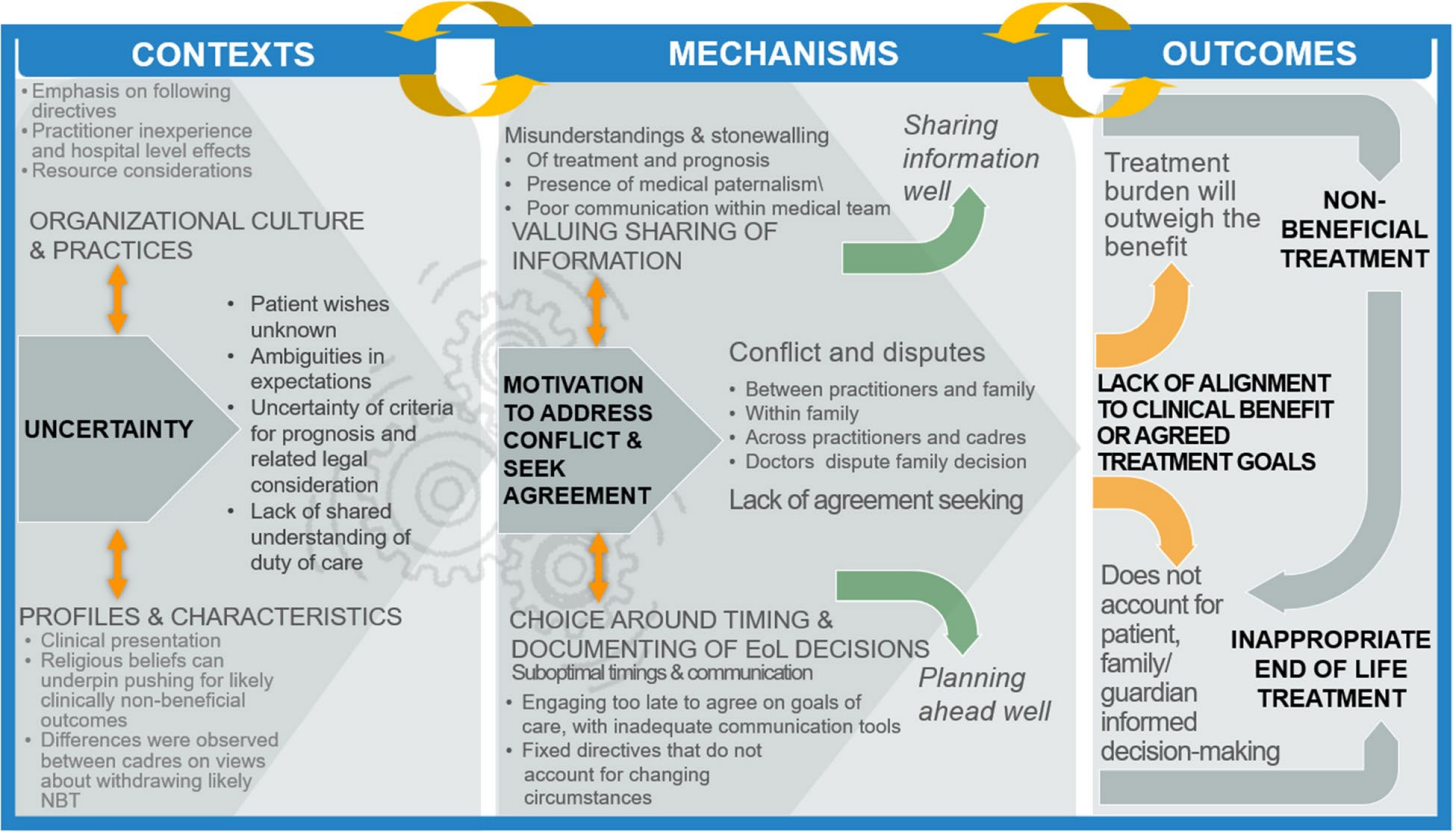
Framework mapping determinants of non-beneficial and inappropriate end-of-life treatment, informing intervention planning and design

Mechanisms to facilitate planning ahead well (*n* = 4) countered these problems. Several studies found that improved advanced care planning, do not resuscitate, and goals of care documentation were needed. For instance, a mixed-methods study found that well-executed documenting about goals of care were significantly associated with lower odds (OR 0.29, 95% CI: 0.10–0.84, *p* = 0.022) of receiving inappropriate treatment at end-of-life [86]. Beyond improved ways of communication and documentation, a couple of studies have also recommended having a primary clinical point-of-contact consistently across the entire hospitalization of a patient could assist in consolidating and streamlining the end-of-life planning process and discussion timings to prevent non-beneficial treatment.

iv) Producing an evidence-based theoretical framework to inform intervention design and future study (Fig. 2)

Based on the findings from objectives a-c, a framework is proposed unpacking Contexts, Mechanisms and related Outcomes to guide further research and intervention design addressing improving non-beneficial and inappropriate end-of-life treatment (Fig. 2). The framework depicts the pathway of the contexts and mechanisms towards the outcomes of interest. Central to this pathway is uncertainty coupled with the need to reach agreements/the role of consensus, since conflict and disputes are major stumbling blocks to end-of-life decision-making. To simplify this “problem”, stonewalling, medical paternalism or a genuine confusion and therefore not sharing of information at all can prevail. Further research and planned interventions should seek to address these issues alongside the timings and documenting of end-of-life decisions such that these are not done either too late nor with fixed directives that do not account for changing circumstances and new information on treatment options, or even personal preferences that may be observed to change over time.

## Discussion

In this study, the importance of both quantitative probability-based and qualitative goal oriented and quality of life-based definitions of non-beneficial treatment and concurrently appropriateness of treatment decisions is emphasized. One of the most commonly cited papers defining “medical futility” by Schneiderman et al. emphasizes the quantitative and qualitative nature of end-of-life treatment [5]. Therein, the authors defined treatments to be “medically futile” if the treatment is known to be probabilistically useless yet still does not cease. The current analysis demonstrates the need to include the appropriateness of treatment in assessments of the benefits of continuing or ceasing to treat, on the grounds that patient autonomy, preferences and medical ethics need explicitly addressing alongside clinical considerations [88]. Accordingly, end-of-life decisions are to be clearly aligned both to clinical benefit as well as agreed treatment goals.

Adding complexity is the consideration of resource consumption and economic burden to the healthcare system [66, 68, 76, 89, 90]. This relates to contexts involving staff burnout and moral distress [75, 91–94] that have been demonstrated to accompany decisions to treat when little or no hope of benefit exists. Emphasizing the distinction between non-beneficial treatment and inappropriate treatment at the end-of-life will serve as a basis to help create a better understanding between policymakers and clinicians by clarifying outcomes of interest of policies. For example, the distinction will allow various stakeholders to understand whether a policy is targeted to reduce inappropriate treatment, non-beneficial treatment, or both. This distinction can also assist in the scientific communication of these concepts. Such dialogues should be seen to spur trickle down effects, improving patient-oriented care. In turn, these effects will trickle upwards to improve healthcare systems, resource management and staff morale more generally.

### Strengths and limitations

Strengths of this review include a drawing upon Realist principles for a more comprehensive and multi-stakeholder perspective. Furthermore, the review had a focused approach on examining this concept in the acute care and non-oncology setting, focusing the review accordingly allowed us to achieve inclusion of a sizable, yet manageable amount of data in which saturation of key findings was achieved. There may be potential application of these findings to the oncology setting, however further synthesis is needed to determine if current findings are transferable. There are several other limitations for this review, such as the focus on only English language articles and only high or middle-high income countries. This hinders the potential generalisability of these findings to other contexts. Future studies should examine the similarities and differences of the framework across different demographic contexts.

## Conclusion

Thus, the framework mapping determinants of non-beneficial and inappropriate end-of-life treatment is proposed. The framework is designed to inform planning of intervention strategies. These strategies should rest on identified mechanisms. The review findings point to the conclusion that the central pathway to improve end-of-life treatment outcomes relies on reducing uncertainty. Finding ways to seek agreement and account for the role of consensus will be key to intervention planning, underpinned by sharing information and planning ahead well.

The proposed framework is positioned as potentially transferable to diverse contexts and suggested mechanisms and pathways should be explored further, tested empirically and updated, such that we learn from the specific to ultimately inform the more general.

## Supplementary Information

**Additional file 1. **PRISMA 2020 checklist and PRISMA 2020 for Abstracts checklist.

**Additional file 2. **Reference list for Tables 2, 3, 4 citations and study characteristics.

## Data Availability

The final included dataset of literature included in this study are shared in the supplementary information file. The screening is transparently reported according to PRISMA guidelines. The screening dataset itself constitutes the intellectual property of the study team, yet the approach and further details of screened data can be shared upon reasonable request.
