# Supplementary material for: A systematic review defining non-beneficial and inappropriate end-of-life treatment in patients with non-cancer diagnoses: theoretical development for multi-stakeholder intervention design in acute care settings
Source: BMC Palliat Care. 2022 Nov 9;21:195. doi: 10.1186/s12904-022-01071-7 (PMC9644578; doi:10.1186/s12904-022-01071-7)
Supplement: Supplementary file 2 — Additional file 2. Reference list for Tables 2, 3, 4 citations and study characteristics. [file 12904_2022_1071_MOESM2_ESM.pdf]

Supplementary file 2: Reference list for Tables 2, 3, 4 citations and study characteristics

Ref #	First Author	Title	Year Published	Setting	Study Design	Sample Size	Participant Characteristics
1	Downar	Nonbeneficial treatment Canada: definitions, causes, and potential solutions from the perspective of healthcare practitioners	2015	Canada, acute care	Quantitative; cross-sectional questionnaire	688 responses	Nursing (74%) and medical staff who provided direct patient care in acute medical wards or ICUs
2	Hsu	The medical futility experience of nurses in caring for critically ill patients	2018	Taiwan, medical center and regional hospital	Qualitative; cross-sectional semi-structured interviews	8 interviews	Nurses with at least 1 year of nursing tenure in the ICU
3	Jox	Medical futility at the end of life: the perspectives of intensive care and palliative care clinicians	2012	Germany, tertiary hospital	Qualitative; case review and semi-structured interviews	17 case consultation reviews and 29 interviews	Of the 29 healthcare professionals, 17 were from intensive care and 12 were from palliative care
4	Rodriguez	Perceptions of patients on the utility or futility of end-of-life treatment	2006	US, medical center	Qualitative; cross-sectional semi-structured interviews	30 elderly patients	Patients were receiving outpatient care from the Veterans Affair medical centre
5	Bolt	Appropriate and inappropriate care in the last phase of life: an explorative study among patients and relatives	2016	The Netherlands, online	Qualitative; cross-sectional online open-ended questionnaires	592 patient and relatives	45 patients and 547 relatives
6	Druwe	Cardiopulmonary resuscitation in adults over 80: outcome and the perception of appropriateness by clinicians	2019	Europe, Israel, Japan, and the USA	Quantitative; cross-sectional questionnaire	611 clinicians	176 doctors, 123 nurses, 312 paramedics
7	Elo	Ethical considerations behind the limitation of cardiopulmonary resuscitation in Hungary--the role of education and training	2005	Hungary, ICU setting	Quantitative; cross-sectional questionnaire	72 doctors	Doctors working in the ICU
8	Halvorsen	Value choices and considerations when limiting intensive care treatment: a qualitative study	2008	Norway, ICU of university hospitals	Qualitative; participant observation and semi-structured In-depth interviews	46 doctors	Anaesthesiologists with decision-making responsibility for intensive care patients and ICU nurses
9	Lambden	Association of perceived futile or potentially inappropriate care with burnout and thoughts of quitting among health-care providers	2019	USA, hospitals	Qualitative; cross-sectional semi-structured interviews	349 clinicians	Attending physicians, residents, nurses, and physician assistants in internal

							medicine, surgery, neurology, or intensive care
10	Monteiro	The decision-making process in families of terminal ICU patients	2019	Brazil, ICU of private hospital	Qualitative; cross-sectional semi-structured interviews	6 family members	Family members of critically ill patients
11	Nelson	Nonbeneficial treatment and conflict resolution: building consensus	2013	USA, hospitals	Quantitative; retrospective evaluation of bioethics consultations	92 case consultations	Cases in which non-beneficial treatment was withdrawn or withheld
12	Heland	Fruitful or futile: Intensive care nurses' experiences and perceptions of medical futility	2007	Australia, ICU setting	Qualitative; cross-sectional semi-structured interviews	7 nurses	Nurses practicing in adult ICUs
13	Neville	Understanding factors contributing to inappropriate critical care: a mixed-methods analysis of medical record documentation	2017	USA, ICU setting	Mixed-methods; qualitative analysis of medical record documentation and quantitative analysis of associated factors	189 patient records	123 patients assessed as receiving inappropriate treatment and 66 patients assessed as receiving appropriate treatment but died within 6 months of ICU admission
14	Schwarzkopf	Perceived nonbeneficial treatment of patients, burnout, and intention to leave the job among ICU nurses and junior and senior physicians	2017	Germany, ICU setting	Quantitative; cross-sectional questionnaire	778 returned and complete questionnaires	ICU nurses (n=628), junior physicians (n=149), and senior physicians (n=70)
15	White	What does “futility” mean? An empirical study of doctors' perceptions	2016	Australia, hospitals	Qualitative; cross-sectional semi-structured interviews	96 doctors	Doctors from three tertiary hospitals in intensive care, palliative care, oncology, renal medicine, internal medicine, respiratory medicine, cardiology, geriatrics, surgery, or emergency specialties
16	Zier	Surrogate decision makers' responses to physicians' predictions of medical futility	2009	USA, hospitals	Mixed-methods; Semi-structured interviews with surrogate decision makers and modified time-trade-off design	50 surrogate decision makers of 50 patients	Surrogate decision makers for patients who were critically ill and incapacitated

17	Bolmsjö	From cure to palliation: agreement, timing, and decision making within the staff	2007	Sweden, hospitals	Quantitative; cross-sectional questionnaire	724 respondents	Physicians (n=415) and nurses (n=309)
18	Anstey	Perceptions of the appropriateness of care in California adult intensive care units	2015	USA, ICU setting	Quantitative; cross-sectional questionnaire	1,363 respondents	Nurses (85%) and doctors (15%) from 52 hospitals with ICUs
19	Anstey	A comparison of the opinions of intensive care unit staff and family members of the treatment intensity received by patients admitted to an intensive care unit: A multicentre survey	2018	Australia, ICU setting	Quantitative; cross-sectional questionnaire	32 patient cases	Staff and surrogate decision-makers of ICU inpatients
20	Bagheri	Experts' attitudes towards medical futility: an empirical survey from Japan	2006	Japan	Quantitative; cross-sectional questionnaire	108 respondents	Members of the Japan Association of Bioethics, with 62% of respondents being healthcare professionals
21	Carter	Factors associated with non-beneficial treatments in end of life hospital admissions: a multicentre retrospective cohort study in Australia	2019	Australia, hospitals	Quantitative; retrospective multicentre cohort study	831 patients	Adult patients who died as inpatients following hospital admission
22	Cauley	Surgeons' perspectives on avoiding nonbeneficial treatments in seriously ill older patients with surgical emergencies: a qualitative study	2016	USA, surgical emergency settings	Qualitative; cross-sectional semi-structured interviews	24 surgeons	Emergency general surgeons
23	Chamberlin	Clinicians' perceptions of futile or potentially inappropriate care and associations with avoidant behaviors and burnout	2019	USA, hospitals	Quantitative; cross-sectional online questionnaire	349 clinicians	Attending physicians, residents, nurses, and physician assistants in internal medicine, surgery, neurology, or intensive care
24	de Menezes	Dysphasia: nursing professionals' perception	2009	Brazil, ICU setting	Qualitative; cross-sectional semi-structured interviews	10 nurses	Nurses working in adult ICU and experiencing dysphasia in their daily practice
25	Gallois	Futile treatment in hospital: doctors' intergroup language	2015	Australia, hospitals	Qualitative; cross-sectional semi-structured interviews	96 doctors	Doctors from three tertiary hospitals in intensive care, palliative care, oncology, renal medicine, internal medicine, respiratory medicine,

							cardiology, geriatrics, surgery, or emergency specialties
26	Goodridge	Caring for critically ill patients with advanced COPD at the end of life: a qualitative study	2008	Canada, ICU setting	Qualitative; cross-sectional focus groups	17 clinicians in three focus groups	15 registered nurses and two respiratory therapists
27	Neville	Differences between attendings' and fellows' perceptions of futile treatment in the intensive care unit at one academic health center: implications for training	2015	USA, ICU setting	Quantitative; prospective observational survey-based study	50 clinicians	36 attending and 14 fellows of ICUs
28	Kadooka	Can physicians' judgments of futility be accepted by patients?: a comparative survey of Japanese physicians and laypeople	2012	Japan	Quantitative; cross-sectional questionnaire	1,535 respondents	1,134 laypeople and 401 physicians
29	Kadooka	A comparative survey on potentially futile treatments between Japanese nurses and laypeople	2013	Japan	Quantitative; cross-sectional questionnaire	1,656 respondents	1,134 laypeople and 522 nurses
30	Longergan	Time-limited trials: a qualitative study exploring the role of time in decision-making on the Intensive Care Unit	2019	England, ICU setting	Qualitative; cross-sectional semi-structured interviews	18 participants	9 doctors, 9 nurses from two ICUs
31	Morris	Shared decision-making in acute surgical illness: the surgeon's perspective	2018	USA, medical centers	Qualitative; cross-sectional semi-structured interviews	20 participants	Practicing surgeons at two large academic medical centers
32	Palda	"Futile" care: Do we provide it? Why? A semi structured, Canada-wide survey of intensive care unit doctors and nurses	2005	Canada, ICU setting	Quantitative; cross-sectional questionnaire	255 responses	141 nurses and 114 physicians from 17 medical centers
33	Piers	Inappropriate care in European ICUs: confronting views from nurses and junior and senior physicians	2014	Belgium, France, Germany, Israel, Italy, Malta, Poland, Portugal, Switzerland, The Netherlands	Quantitative; cross-sectional questionnaire	1,651 responses	1,218 nurses, 180 junior physicians, 227 senior physicians, 26 unknown

34	Rostami	Perception of futile care and caring behaviors of nurses in intensive care units	2017	Iran, ICU setting	Quantitative; cross-sectional questionnaire	181 nurses	Nurses with at least 1 year working experience in the ICU and full-time employment in the ICU
35	Shahryari	Attitudes of physicians to futile treatment at the end of life care	2013	Iran, medical centers	Quantitative; cross-sectional questionnaire	200 doctors	Physicians serving in medical educational centres
36	Sibbald	Perceptions of "futile care" among caregivers in intensive care units	2007	Canada, ICU setting	Qualitative; cross-sectional semi-structured interviews	44 interviews	14 physician directors, 16 nurse managers, 14 respiratory therapists from 16 ICUs
37	Singal	A prospective determination of the incidence of perceived inappropriate care in critically Ill patients	2014	Canada, ICU setting	Quantitative; prospective observational survey-based study	6,558 surveys representing 294 patients	Surveys completed by respective bedside nurse, charge nurse and first-call intensivist
38	Small	Dying, death and bereavement: a qualitative study of the views of carers of people with heart failure in the UK	2009	UK	Qualitative; cross-sectional semi-structured interviews	20 caregivers	Family carers of deceased heart failure patients
39	Wilson	Prevalence of disagreement about appropriateness of treatment between ICU patients/surrogates and clinicians	2019	US and Hungary, ICU setting	Quantitative; prospective observational survey-based study	151 patients	Patients had patient and/or surrogate survey completed
40	Yekefallah	Nurses' experiences of futile care at intensive care units: a phenomenological study	2015	Iran, ICU setting	Qualitative; cross-sectional semi-structured in-depth interviews and participant observation	25 nurses	Nurses working in the ICU in teaching hospitals
41	Pavlish	Nursing priorities, actions, and regrets for ethical situations in clinical practice	2011	USA	Qualitative; cross-sectional open-ended questionnaire	91 nurses	Nurses who registered to attend a nursing ethics conference
42	Batten	What does the word "treatable" mean? Implications for communication and decision-making in critical illness	2019	USA	Qualitative; cross-sectional semi-structured interviews	48 participants	24 nonphysicians (patients and community members) and 24 physicians
43	Calvin	The cardiovascular intensive care unit nurse's experience with end-of-life care: a qualitative descriptive study	2009	USA, ICU setting	Qualitative; cross-sectional semi-structured interviews	19 participants	Nurses working in a cardiovascular ICU at a tertiary care teaching hospital

44	Robichaux	Practice of expert critical care nurses in situations of prognostic conflict at the end of life	2006	USA, ICU setting	Qualitative; cross-sectional semi-structured interviews	21 participants	Critical nurses nominated as experts by their colleagues
45	Ruopp	Questioning care at the end of life	2005	USA, hospitals	Mixed-methods; qualitative interviews and open-ended questions, quantitative questionnaire	188 participants	188 physicians with a subsample of 75 physicians narratives for qualitative data analysis
46	Beck	A "little bit illegal"? Withholding and withdrawing of mechanical ventilation in the eyes of German intensive care physicians	2008	Germany, ICU setting	Qualitative; cross-sectional semi-structured interviews	28 participants	Physicians working in ICU wards
47	Ntantana	The impact of healthcare professionals' personality and religious beliefs on the decisions to forego life sustaining treatments: an observational, multicentre, cross-sectional study in Greek intensive care units	2016	Greece, ICU setting	Quantitative; cross-sectional questionnaire	469 participants	149 doctors and 320 nurses
48	Aghabarary	Reasons behind providing futile medical treatments in Iran	2017	Iran, hospitals	Qualitative; cross-sectional semi-structured interviews	30 participants	21 nurses and 9 medical specialists from teaching hospitals
49	Hayes	Clinical model for ethical cardiopulmonary resuscitation decision-making	2012	Australia	Qualitative; cross-sectional semi-structured interviews	33 participants	Doctors that were expected to have considerable experience in making CPR decisions
50	Glogowska	"Sometimes we can't fix things": a qualitative study of health care professionals' perceptions of end of life care for patients with heart failure	2016	UK	Qualitative; cross-sectional semi-structured interviews	24 participants	Healthcare professionals involved in care of heart failure patients
51	Robinson	Implementing a resuscitation policy for patients at the end of life in an acute hospital setting: qualitative study	2007	Ireland, hospitals	Qualitative; cross-sectional semi-structured interviews	16 participants	7 nurses and 9 doctors involved in CPR decision-making
52	Willmott	Reasons doctors provide futile treatment at the end of life: a qualitative study	2016	Australia, hospitals	Qualitative; cross-sectional semi-structured interviews	96 doctors	Doctors from three tertiary hospitals in intensive care, palliative care, oncology, renal medicine, internal medicine, respiratory medicine,

							cardiology, geriatrics, surgery, or emergency specialties
53	Dzeng	Moral distress amongst American physician trainees regarding futile treatments at the end of life: a qualitative study	2015	USA, hospitals	Qualitative; cross-sectional semi-structured interviews	22 participants	Internal medicine residents and fellows from academic medical centers
54	Moore	Age as a factor in do not attempt cardiopulmonary resuscitation decisions: A multicentre blinded simulation-based study	2015	UK, hospitals	Quantitative; cross-sectional questionnaire	291 participants	Consultant physicians, surgeons and anaesthetists
55	Noureddine	Assessment of cardiopulmonary resuscitation practices in emergency departments for out-of-hospital cardiac arrest victims in Lebanon	2016	Lebanon, emergency department	Quantitative; cross-sectional questionnaire	75 participants	Physicians working in emergency departments
56	Piers	Perceptions of appropriateness of care among european and israeli intensive care unit nurses and physicians	2011	9 European countries and Israel, ICU setting	Quantitative; cross-sectional questionnaire	1,651 participants	ICU nurses and physicians providing bedside care
57	So	Questionnaire survey on medical futility and termination of resuscitation in cardiac arrest patients among emergency physicians in Hong Kong	2019	Hong Kong, emergency department	Quantitative; cross-sectional questionnaire	140 participants	Emergency medicine physicians
58	Svantesson	Nurses' and physicians' opinions on aggressiveness of treatment for general ward patients	2006	Sweden, hospitals	Mixed-methods; cross-sectional structured interviews and evaluation of nurse-physician agreement	224 interviews regarding 714 patients	Nurses and physicians, with paired nurse-physician interviews
59	Trankle	Is a good death possible in Australian critical and acute settings?: physician experiences with end-of-life care	2014	Australia, ICU and hospital setting	Qualitative; cross-sectional semi-structured interviews	13 physicians	Specialist physicians with relevant experience in end-of-life care
60	Kirby	The role and significance of nurses in managing transitions to palliative care: a qualitative study	2014	Australia, hospitals	Qualitative; cross-sectional semi-structured interviews	20 nurses	Hospital-based nurses who regularly work with patients at the point of referral and in managing transitions to palliative care

Supplementary file 2: Reference list for Tables 2, 3, 4 citations and study characteristics

61	Neville	Concordance of nurses and physicians on whether critical care patients are receiving futile treatment	2015	USA, ICU setting	Mixed-methods; cross-sectional focus groups and patient survival assessments	6,254 shared assessments on 1,086 patients	Nurses and physicians working in critical care
62	Le	Care of the dying in Australia's busiest hospital: benefits of palliative care consultation and methods to enhance access	2010	Australia, hospitals	Mixed-methods; retrospective chart review and cross-sectional semi-structured interviews	171 records and 27 interviews	Senior clinicians from most departments and disciplines
63	Bulow	Are religion and religiosity important to end-of-life decisions and patient autonomy in the ICU? The Ethicatt study	2012	6 European countries, ICU and hospital setting	Quantitative; cross-sectional questionnaire	1,268 participants	304 physicians, 386 nurses, 248 patients and 330 family members
64	Malhotra	Instability in end-of-life care preference among heart failure patients: secondary analysis of a randomized controlled trial in Singapore	2020	Singapore	Quantitative; secondary analysis with prospective questionnaires	200 participants	Patients with heart failure
65	Efstathiou	Compassionate care during withdrawal of treatment: a secondary analysis of ICU nurses' experiences	2018	UK, ICU setting	Qualitative; cross-sectional semi-structured interviews	13 nurses	Nurses from ICUs within a large hospital
66	Micallef	Level of agreement on resuscitation decisions among hospital specialists and barriers to documenting do not attempt resuscitation (DNAR) orders in ward patients	2011	Australia, emergency department	Quantitative; prospective observational survey-based study	1,458 patients	Ward patients during medical emergency team calls
